# Downregulation of the neonatal Fc receptor expression in non-small cell lung cancer tissue is associated with a poor prognosis

**DOI:** 10.18632/oncotarget.10074

**Published:** 2016-06-15

**Authors:** Emilie Dalloneau, Nadine Baroukh, Konstantinos Mavridis, Agnès Maillet, Fabien Gueugnon, Yves Courty, Agnès Petit, Thomas Kryza, Maguy Del Rio, Serge Guyetant, Diana Carolina Cadena Castaneda, Christine Dhommée, Christophe Arnoult, Andreas Scorilas, Valérie Gouilleux-Gruart, Nathalie Heuzé-Vourc'h

**Affiliations:** ^1^ Université François Rabelais, UMR 1100, Tours, France; ^2^ INSERM, Centre d'Etude des Pathologies Respiratoires, UMR 1100, Tours, France; ^3^ Université François Rabelais de Tours, CNRS, GICC UMR 7292, Tours, France; ^4^ Department of Biochemistry and Molecular Biology, University of Athens, Panepistimiopolis, Athens, Greece; ^5^ Institute of Health and Biomedical Innovation, Translational Research Institute, Queensland University of Technology, Brisbane, Queensland, Australia; ^6^ IRCM, Institut de Recherche en Cancérologie de Montpellier, INSERM U1194, Montpellier, France; ^7^ CHRU de TOURS, Laboratoire d'Immunologie, Tours, France

**Keywords:** FcRn, non-small cell lung cancer, prognosis, marker, antitumor immunity

## Abstract

Lung cancer is the leading cause of cancer-related death worldwide. Although the recommended tumor, node and metastasis (TNM) classification and stage determination are important to select therapeutic options for patients with non-small cell lung carcinoma (NSCLC), additional molecular markers are required to indicate the prognosis, in particular within a specific stage, and help with the management of patients.

Because neonatal Fc receptor (FcRn) has recently been involved in colon cancer immunosurveillance, we measured its expression in non-cancerous and NSCLC lung tissues and evaluated its prognostic value in overall survival for patient with NSCLC. FcRn expression was determined at both mRNA and protein levels on cancerous and adjacent non-cancerous tissues from 80 NSCLC patients. In NSCLC, FcRn was mainly found in resident and tumor infiltrating immune cells. The corresponding mRNA and protein were significantly less abundant in lung tumor than non-cancerous tissue. Moreover, analysis of our cohort and datasets from the public data bases show that *FCGRT* mRNA down-regulation is a robust and independent, unfavorable predictive factor of NSCLC patient survival. We conclude that *FCGRT* mRNA expression may be a useful additional marker for immunoscoring, reflecting tumor immune system, and help in the decision-making process for NSCLC patients.

## INTRODUCTION

Lung carcinogenesis is complex, involving both neoplastic cells and the tumor microenvironment. Today, the complex interplay between the immune system and lung tumor is well documented [1]. The quality of antitumor immune responses relies on lymphocytes, macrophages and granulocytes. Cytotoxic CD8^+^ T lymphocytes (CTL), CD4^+^ T lymphocytes, B-lymphocytes and natural killer (NK)/natural killer T (NKT) cells, are known to play a major role in the cytotoxic attack against tumor cells. The success of this attack depends partly on an effective antigen presentation by tumor cells and antigen presenting cells (APC), including macrophages and dendritic cells (DC). As documented in various solid tumors, the nature, the density and the location of immune cells correlate with cancer patient prognosis. In non-small cell lung cancer (NSCLC), mature DC and follicular B-cell density, and infiltration of CD8^+^ T cells correlate with a better clinical outcome [2, 3]. Interestingly, molecular changes, related to antitumor immune response, may also constitute prognosis biomarkers. For example, high expression of calreticulin, which participates to the immunogenic cell death and antigenicity of tumor cells, constitutes a favorable molecular prognosis biomarker in NSCLC [4].

FcRn belongs to the family of receptors for the Fc portion of IgG and is encoded by *FCGRT*. FcRn is expressed in many cells and tissues throughout life. It is found in endothelial and epithelial cells from various organs (including placenta, lung, intestine and brain) where it participates in the recycling and transcytosis of IgG [5, 6]. This contributes to the long half-life of IgGs in biological fluids and their distribution in the human body [5, 7–9]. Aside from these well-known biological functions, FcRn is also involved in the humoral immune response: present in the epithelia of mucosa, FcRn is important for the host immune response against both bacteria and viruses [10–13]. FcRn allows virus-specific IgG to bind with pathogens in epithelial cell endosomes where it neutralizes virus [13]. FcRn in immune cells, in particular dendritic cells, macrophages, monocytes [14] and neutrophils [15], is involved in antigen presentation and cross-presentation by dendritic cells [16, 17]. Recently, FcRn has been shown to play a pivotal role in anti-tumor immunity. Indeed, FcRn mediated tumor protection through DC activation of endogenous tumor-reactive CD8^+^-T cells via the cross-presentation of IgG complexed antigens, in a colorectal cancer model, using *fcgrt* knock-out mice [18]. Moreover, the density of FcRn-expressing DC correlated with CD8^+^ T cell numbers and predicted improved prognosis in human colorectal carcinoma.

Based on these results, we conducted a retrospective study in several NSCLC cohorts, to evaluate the prognosis value of FcRn expression in lung cancer: the lung is one of the major organs expressing FcRn and lung cancer is the leading cause of cancer-related mortality [19]. Herein, we showed for the first time that *FCGRT* mRNA is down-regulated in non-small cell lung carcinoma (NSCLC) patients and that *FCGRT* mRNA levels in both NSCLC and adjacent non-cancerous tissues are independently positively correlated with prognosis. We found that FcRn was mainly expressed by resident and tumor-infiltrating immune cells, in the lung, indicating that *FCGRT* mRNA level might reflect lung antitumor immune response.

## RESULTS

### *FCGRT* mRNA is down-regulated in NSCLC tissue

**Figure 1:**
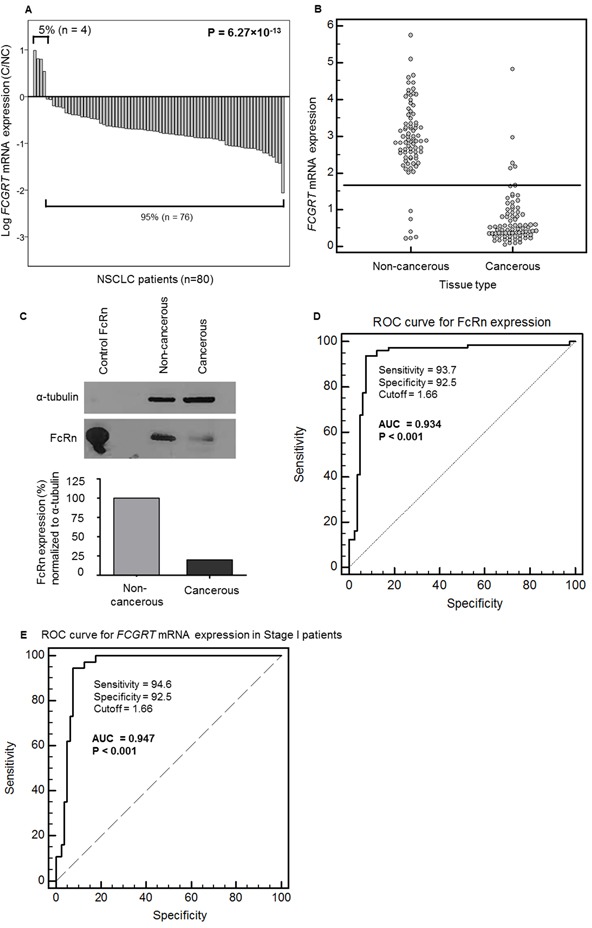
A. Ratios of *FCGRT* mRNA in cancerous (C) / non-cancerous (NC) in paired tissues from the NSCLC patients (n=80; P = 6 27×10^−13^, Wilcoxon Signed Ranks test). **B.** Distribution of FCGRT mRNA levels in non-cancerous and cancerous tissues (n=80; P = 2.54×10^−21^, Mann-Whitney test). **C.** Representative image of FcRn protein revealed by western blotting in a pool of 10 patients with matched cancerous and adjacent non-cancerous tissue. Recombinant human FcRn protein was loaded as a positive control. Signals were quantified with ImageJ and normalized to that for α-tubulin. **D.** ROC curve analysis for FCGRT mRNA level in cancerous and non-cancerous lung tissue samples. n= 80, AUC = 0.934, SE = 0.024, 95% CI = 0.884 – 0.967, P < 0.0001, calculations according to DeLong *et al.*, 1988. Youden index J = 0.863 (95% CI = 0.775 – 0.925, BCa bootstrap interval, 1000 iterations). **E.** ROC curve analysis for FCGRT mRNA levels in stage I cancerous and in non-cancerous tissues (n=37; AUC = 0.947, 95% CI = 0.905 – 0.989, P < 0.001, calculations according to DeLong *et al.*, 1988. Youden index J = 0.871 (95% CI = 0.771 – 0.938, BCa bootstrap interval, 1000 iterations).

*FCGRT* mRNA was assayed by qRT-PCR in cancerous and non-cancerous samples from patients with NSCLC (Figure 1A). Mean *FCGRT* mRNA levels were significantly lower in the cancerous (mean ± SE = 0.727 ± 0.080) than non-cancerous (mean ± SE = 2.95 ± 0.12) tissue. *FCGRT* mRNA levels were lower in 95% (76/80) of the NSCLC than paired non-cancerous tissues (P < 0.001). The median *FCGRT* mRNA values were 6-fold lower in the cancerous than non-cancerous samples (median tumor = 0.482, median adjacent normal tissue = 2.89; P < 0.001) (Figure 1B). Similarly, western blotting of pooled protein extracts from different patients, chosen randomly, showed that the amount of FcRn (normalized to α-tubulin) was much lower in cancerous than non-cancerous tissue (Figure 1C). Using a cut-off value of 1.66 expression units, the relative *FCGRT* mRNA levels in cancerous and non-cancerous tissues showed a sensitivity of 93.7% and a specificity of 92.5%, (Figure 1D). ROC analysis of *FCGRT* mRNA levels (Figure 1D) produced a notable AUC of 0.934 (SE = 0.024, 95% CI = 0.884 – 0.967, P < 0.001). At fixed sensitivities of 90.0% and 95.0%, specificity values were 92.50% and 87.50%, respectively. At fixed specificities of 80.0% and 90.0%, sensitivity values were 97.50% and 93.75%, respectively. Findings for stage I specimens were similar: the difference in expression was 5-fold (P < 0.001), and the corresponding ROC curve analysis generated an AUC of 0.947 (95% CI = 0.905 – 0.989, P < 0.001) (Figure 1E). Similarly, the discriminatory capacity of FcRn was also robust for stage>I patients (AUC = 0.923, 95% CI = 0.862 – 0.983, P <0.001). Using a cut-off value at 1.66 expression units, we can distinguish cancerous tissues from non-cancerous with a strong efficiency; this holds true even for early stage (stage I) patients for whom *FCGRT* mRNA expression analysis offers important differential diagnostic information indicating that it could enhance the accuracy of trans-bronchial needle aspirations or small biopsies. No significant association was found between *FCGRT* mRNA levels in cancerous tissues and clinic-pathological features (Supplementary Table S1).

### Expression of FcRn in NSCLC patients is mainly attributed to immune cells

**Figure 2:**
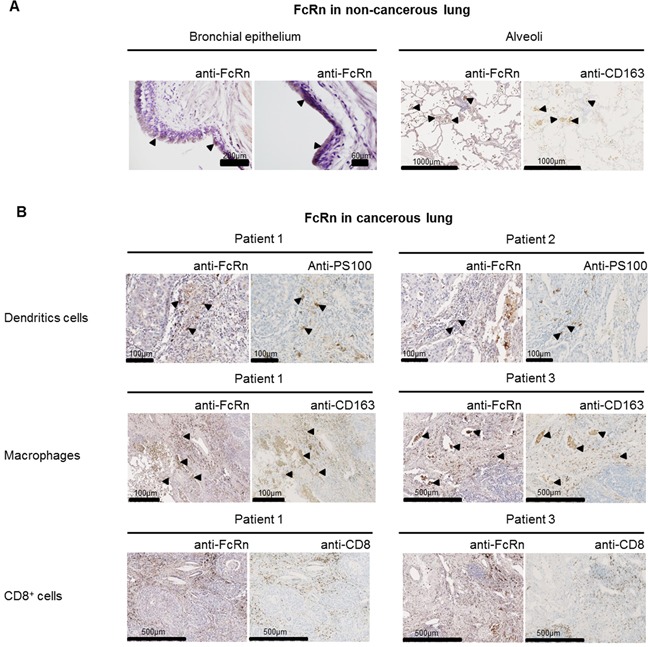
Expression of FcRn in non-cancerous A. and cancerous B. serial lung sections from a small set of patients (n=8) **(A)** FcRn expression was very week in bronchial epithelial cells (left panel) and marked in alveolar macrophages (right panel) in the non-cancerous lung. **(B)** In cancerous tissue, the staining revealed that FcRn is expressed in interstitial stromal cells including DCs (PS100), macrophages (CD163) (arrowheads indicate areas of colocalization) but no CD8^+^ T cells (CD8). A very weak staining was also observed in carcinomatous cells. Pictures from 3 patients were selected as they are representative of the different staining.

The distribution of FcRn protein has been studied in the normal lungs of various species and is restricted to bronchial epithelial cells and alveolar macrophages in humans [20]. NSCLC originate mainly from epithelial bronchial cells (and in some cases from epithelial alveolar cells), so we tested for FcRn expression by immunohistochemistry (see supplemental results for IHC validation) in a small set of cancerous and non-cancerous lung tissues. We detected FcRn in alveolar macrophages and at very low levels in the bronchial epithelium of the non-cancerous tissue (Figure 2). In tumor samples, it was very low in neoplastic cells while mainly detected in large cells, located in immune islets, in the stromal and peri-vascular compartment (Figure 2). Staining for CD8 (CD8^+^ T cells), CD163 (macrophages) or PS100 (dendritic cells) on serial sections revealed that they were macrophages and dendritic cells (Figure 2), as previously described by Baker *et al*. in human colorectal carcinomas [18].

### Prognostic value of *FCGRT* mRNA in NSCLC patients

#### *FCGRT* mRNA in cancerous tissues is associated with a favorable prognosis

**Figure 3:**
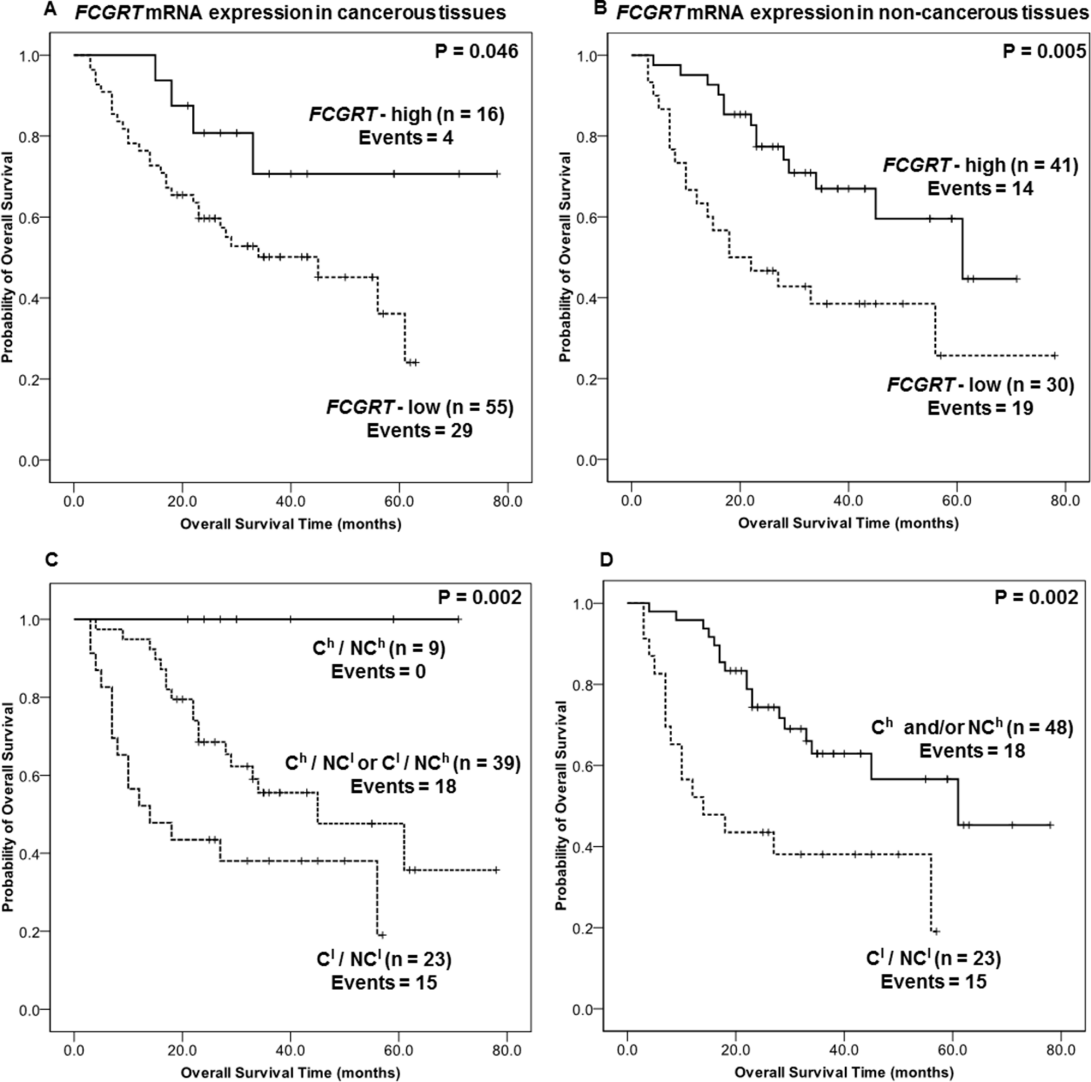
A. Kaplan-Meier overall survival analyses for FCGRT mRNA level in cancerous tissue from NSCLC patients (n=71) Cut-off value = 0.888 (74^th^ percentile of FCGRT mRNA abundance in cancerous samples). **B.** Kaplan-Meier overall survival analysis for FCGRT mRNA level in non-cancerous tissue from NSCLC patients (n=71). Cut-off value = 2.82 (43^th^ percentile of *FCGRT* expression in normal tissues). **C-D.** Kaplan-Meier overall survival analysis for *NSCLC* patients stratified according to *FCGRT* mRNA level in cancerous and non-cancerous (n=71). **(C)** C^h^ / NC^h^: Patients with high *FCGRT* mRNA levels in both tissue parts. C^h^ / NC^l^ or C^l^ / NC^h^: Patients with high *FCGRT* mRNA expression only in the cancerous or the non-cancerous tissue, respectively. C^l^ / NC^l^: Patients with low *FCGRT* mRNA levels in both tissue parts (n=71). **(D)** C^h^ and/or NC^h^: Patients with high *FCGRT* mRNA levels in at least one tissue part (cancer and/or non-cancerous tissues n=71). C^l^ / NC^l^: Patients with low *FCGRT* mRNA levels in both tissue parts. Cut-off values as described above.

Baker *et al*. showed that the frequency of specific FcRn-positive cells correlated with survival in colorectal carcinoma [18], so we evaluated the predictive value of testing for FcRn in cancerous and non-cancerous lung tissues. The predictive value of FcRn for NSCLC patient survival was evaluated by analyzing *FCGRT* mRNA expression (high or low) in cancer tissues. The survival of NSCLC patients classified as *FCGRT*-high (62.0 months (SE = 6.9)) was better than that of *FCGRT*-low patients (37.3 months (SE = 3.3); P = 0.046 Kaplan-Meir analysis) (Figure 3A); this was also shown by univariate Cox regression analysis (HR = 0.362, 95% CI= 0.127 – 1.03, P = 0.057) (Table 1). Multivariate Cox regression analysis, adjusted for significant clinicopathological variables, identified *FCGRT* mRNA expression in the cancerous tissues as an independent indicator of favorable prognosis for NSCLC patients (HR = 0.332, 95% CI = 0.112 – 0.983, P = 0.047) (Table 1).

**Table 1:** Cox regression overall survival analyses at the univariate and multivariate levels (n=71)

Univariate Analysis					Multivariate Analysis				
Variable	HR	95% CI	*P* value	Bootstrap *P* value	Variable	HR	95% CI	*P* value	Bootstrap *P* value
***FCGRT* mRNA level in cancerous tissue**					***FCGRT* mRNA level in cancerous**				
Low (l) (n=55)	1.00				Low	1.00			
High (h) (n=16)	0.362	0.127 – 1.03	0.057	0.041	High	0.332^a^	0.112 – 0.983	**0.047**	0.042
						0.267^b^	0.089 – 0.806	0.019	0.013
***FCGRT* mRNA level in non-cancerous tissue**					***FCGRT* mRNA level in non-cancerous tissue**				
Low (n=30)	1.00				Low	1.00			
High (n=41)	0.384	0.192 – 0.769	**0.007**	0.003	High	0.323^a^	0.154 – 0.678	**0.003**	0.002
						0.339^b^	0.162 – 0.709	0.004	0.008
***FCGRT* mRNA levels in both tissue types**					***FCGRT* mRNA levels in both tissue type**				
C^l^/NC^l^ (n=23)	1.00				C^l^/NC^l^	1.00			
C^h^and/or NC^h^(n=48)	0.355	0.177 – 0.714	0.004	0.003	C^h^and/or NC^h^	0.273^a^	0.129 – 0.577	**0.001**	0.001
						0.263^b^	0.124 – 0.556	<0.001	0.002
**Stage** (n=71)	1.22	1.04 – 1.44	0.015	0.009					
**Histotype**									
SCC (n=29)	1.00								
ADC (n=42)	1.23	0.605 – 2.50	0.566	0.570					
**Age** (n=71)	0.992	0.961 – 1.02	0.596	0.565					
**Metastasis**									
No (n=57)	1.00								
Yes (n=12)	2.37	1.09 – 5.17	0.030	0.012					

a Multivariate model adjusted for stage, histotype, age (model a).

b Multivariate model adjusted for metastasis status, histotype, age (model b).

#### *FCGRT* mRNA in non-cancerous tissues is also an independent predictor of survival for NSCLC patients

Taking into consideration the recently described role of FcRn in the anti-tumor immune response [18], and the prognostic gene expression signatures that can be derived from tumor-adjacent tissue parts as previously reported for several human malignancies [21, 22], we sought to evaluate the predictive value of *FCGRT* mRNA expression in non-cancerous tissues obtained from NSCLC patients. High *FCGRT* mRNA levels in the non-cancerous specimens were associated (P = 0.005) with favorable prognosis (Figure 3B), whereas patients with low *FCGRT* mRNA expression were 2.6 times more likely to die of the disease, as indicated by univariate analysis (P=0.007; Table 1). Furthermore, *FCGRT* mRNA expression in non-cancerous tissues was found to constitute a strong independent predictor of favorable overall survival outcome in NSCLC patients (HR = 0.323, 95% CI = 0.154 – 0.678, P = 0.003 Table 1). Altogether those results showed that *FCGRT* mRNA higher levels in cancerous and, intriguingly non-cancerous tissue are associated with a favorable outcome and provide prognosis information independently of other clinic-pathological parameters.

#### Assessment of *FCGRT* mRNA in both the tumor and non-cancerous tissues can stratify NSCLC patients according to overall survival

We further stratified NSCLC patients, according to *FCGRT* mRNA levels in both cancerous (C) and non-cancerous (NC) tissue. Overall survival periods were longer for high (h) than low (l) *FCGRT* mRNA levels in both cancerous and non-cancerous tissues (P = 0.002) (Figure 3C–3D). Strikingly, none of the “double high” (C^h^ /NC^h^) patients died during the follow-up period, whereas the survival probabilities progressively worsened over time for patients with one or both tissue types scored as *FCGRT*-low (Figure 3C). The findings were similar when the patients were grouped into those with high FcRn expression in at least one tissue type (C^h^ and/or NC^h^) and those with low *FCGRT* expression in both tissue types (C^l^/NC^l^): the latter group showed significantly worse outcomes (P = 0.002) (Figure 3D).

Thus, analysis of *FCGRT* mRNA expression in both tissues types can provide significant and robust prognostic information about NSCLC patients as shown by univariate Cox regression (HR = 0.355, 95% CI = 0.177 – 0.714, P = 0.004) (Table 1), which is independent of the currently used conventional indicators and important clinico-pathological variables as proven by multivariate survival analysis (HR = 0.273, 95% CI = 0.129 – 0.577, P = 0.001) (Table 1).

#### Prognostic value of *FCGRT* mRNA in early stage and in metastasis-free NSCLC patients

We analyzed the prognostic performance of *FCGRT* mRNA levels in subgroups of NSCLC patients conventionally classified as “lower-risk”. Kaplan-Meier survival analysis within the early stage (I/II) patient subgroup indicated that high *FCGRT* mRNA levels retained their association with favorable outcome; this was statistically significant for non-cancerous tissue (P = 0.035). Early stage patients with high levels of *FCGRT* mRNA in at least one tissue (C^h^ and/or NC^h^) survived longer than patients with low levels of *FCGRT* mRNA in both tissues (C^l^ / NC^l^) (P= 0.055). Similar observations were made for the non-metastatic patients, as *FCGRT* mRNA expression again endowed NSCLC patients with enhanced overall survival intervals; this held true for the determination of *FCGRT* mRNA expression in non-cancerous tissue parts (P = 0.007) and the comparisons between NSCLC patients with at least one tissue part with a high expression *versus* patients with both tissue having a low expression of *FCGRT* mRNA (P= 0.008) (see Supplementary Table S2).

Multivariate analysis confirmed the independent prognostic information from *FCGRT* mRNA expression levels in normal and cancerous tissues, further demonstrating the similar yet discrete clinical significance of these assessments (data not shown).

#### Prognostic value of *FCGRT* mRNA expression is validated in others cohorts with NSCLC

**Figure 4:**
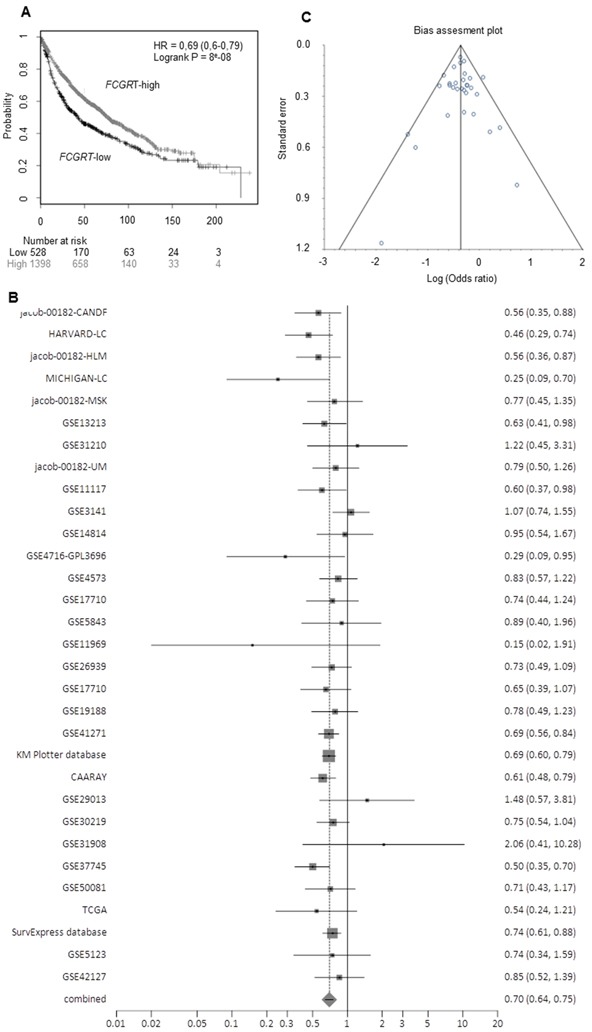
A. Kaplan-Meier overall survival curves based on *FCGRT* expression (high vs low), as assessed by the KM plotter expression analysis data **B.** Forest plot (random effects method) of the hazard ratio for *FCGRT* expression and overall survival in lung cancer based on data obtained by the PrognoScan, PROGgeneV2, Kaplan Meier Plotter and SurvExpress platforms. **C.** Bias assessment plot for trials considered in the meta-analysis.

In order to reinforce our findings regarding the prognostic significance of *FCGRT* mRNA expression in lung cancer, we performed *in silico* analysis of FcRn expression. Firstly, we analyzed microarrays studies extracted from the Oncomine database [23]. The results showed that *FCGRT* mRNA down regulation was also associated with poor survival of NSCLC patients in several datasets, supporting the results of our qRT-PCR study (Tables 2 and 3). Secondly, we used Affymetrix microarray expression data from lung cancer patients and analyzed *FCGRT* mRNA expression as determined by probe set 218831_s_at, based on the online “Kaplan Meier Plotter” tool [24]. The significant association of *FCGRT* mRNA expression with favorable overall survival of lung cancer patients that we observed, was validated using this independent dataset of 1,926 lung cancer samples (Figure 4A, see Supplementary Table S3) at the univariate (HR = 0.69, 95% CI = 0.6 – 0.79, P = 8×10^−8^), and multivariate levels after adjustment for stage and tumor histotype (HR = 0.69, 95% CI = 0.55 - 086, P = 0.0009) (see Supplementary Table S3). Finally to validate these results, we performed a meta-analysis on data from publicly available expression analysis platforms: Kaplan Meier Plotter [24], PrognoScan [25], PROGgeneV2 [26] and SurvExpress [27]. *FCGRT* mRNA expression was strongly associated with favorable overall survival of lung cancer patients as indicated by the pooled HR from n=31 studies/databases HR= 0.70 (95% CI = 0.65-0.74), P < 0.0001. In 28/31 studies the HRs were <1 (indicating association with favorable prognosis) and 12/31 were individually statistically significant, including large cohorts from the Kaplan Meier plotter database (N=1926), the SurvExpress platform (N=1044) and the CAARAY, NCI (N=468) (Figure 4B, see Supplementray Table S4). Moreover, no statistical heterogeneity was observed (Cochran Q = 33.08, df = 30, P = 0.3191, I^2^ = 9.3%, 95% CI = 0-42%), neither statistically significant bias (Begg-Mazumdar: Kendall's tau = 0.0409 P = 0.7616, Egger: bias = −0.04965, 95% CI = −0.790–0.691, P = 0.8918) (Figure 4C, see Supplementary Table S4). The absence of statistically significant heterogeneity and bias are indicators of the sufficient quality of the meta-analyses and the validity of the deriving conclusions.

**Table 2:** Association between survival status and down-regulation of *FCGRT* mRNA expression in some human lung cancer datasets extracted from Oncomine

Clinical parameters significantly associated to reduced *FCGRT* mRNA levels in human Lung cancer datasets	n	p-value	Dataset Reference
Lung Adenocarcinoma, Dead at 1 Year	76	0.03	Beer Lung, Nat Med, 2002
	21	0.014	Garber Lung, Proc Natl Acad Sci U S A, 2001
Large Cell Lung Carcinoma, Dead at 3 Years	10	0.016	Zhu Lung, J Clin Oncol, 2010
Large Cell Lung Carcinoma, Dead at 5 Years	12	0.012	Hou Lung, PLoS One, 2010
	10	0.016	Zhu Lung, J Clin Oncol, 2010
Squamous Cell Lung Carcinoma, Dead at 5 Years	10	0.038	Hou Lung, PLoS One, 2010

**Table 3:** Associations between *FCGRT* mRNA expression and survival status of NSCLC patients at 1 year after surgery (Tours' hospital cohort)

*FCGRT* expression	Number of patients (%)		P*
	*1-year survival*		
**Cancerous tissue**	Dead	Alive	
Low (l)	13 (100.0)	42 (72.4)	0.032
High (h)	0 (0.0)	16 (27.6)	
**Non-cancerous tissue**	Dead	Alive	
Low	11 (84.6)	19 (32.8)	0.001
High	2 (15.4)	39 (67.2)	
**Combined**	Dead	Alive	
C^l^ / NC^l^	11 (84.6)	12 (20.7)	<0.001
C^h^ and/or NC^h^	2 (15.4)	46 (79.3)	

* Fisher's exact test

## DISCUSSION

The lungs are one of the major organs expressing FcRn and a common site of carcinogenesis. In this study, we showed a significant decrease of FcRn expression, at both the mRNA and protein levels, in the lung cancerous compared to the lung non-cancerous tissues from NSCLC patients. This discriminative value of *FCGRT* mRNA expression between cancer lesions from non-cancerous tissue parts is high, even for early stage patients (AUC = 0.947). This result is in line with the recent evidences demonstrating a central role of FcRn in anti-tumor immune-surveillance [18]. It also raises questions on the role of FcRn in non-cancer lung diseases, since lower levels of *FCGRT* mRNA were also described in bronchiectasis and lung fibrosis, correlating with the extent of lung abnormalities [28].

Herein, we also found that *FCGRT* mRNA levels were associated with a favorable outcome and provided prognostic information independently from important clinic-pathological parameters, when it was assessed in the cancer or, intriguingly, in the non-cancerous tissue part. In fact, *FCGRT* mRNA expression, especially when assessed in the non-cancerous tissue part has prognostic relevance even for the “lower-risk” early stage (P = 0.035) and metastasis-free NSCLC patients (P = 0.007). Interestingly, multivariate analysis also corroborated the similar yet discrete clinical significance of these assessments (data not shown). Overall our findings are consistent with the accumulating evidences showing molecular alterations of the normal-appearing tissue adjacent to tumor lesions and their impact in the stepwise progression to lung cancer [29]. Considering the role of FcRn in immunosurveillance and tumorigenesis, the decrease in *FCGRT* mRNA expression might be an important biomarker of the molecular changes promoting lung carcinogenesis and tumor progression. It may help with decision-making for NSCLC management and yield important advances for the development of novel chemoprevention strategies.

When analyzing the combined non-cancerous and cancerous expression of *FCGRT* mRNA, we noticed a progressive deterioration of overall survival periods with the decrease presence of *FCGRT* mRNA in non-cancerous and cancerous tissue parts. None of the patients classified as *FCGRT* mRNA *-*high for both tissue parts died within the study's follow-up period, whereas the survival probabilities worsened when moving to patients with one tissue part and both tissue parts showing low expression. This observation extends the tumor-protective role of FcRn in the lungs. The combined analysis of *FCGRT* mRNA expression can provide significant and robust prognostic information regarding the overall survival of NSCLC patients. The robustness of our findings is clearly reinforced by the fact that same results were found in other independent and international cohorts. It was found to be independent of the currently used conventional indicators and important clinic-pathological parameters. Moreover, these results were confirmed by the meta-analysis realized on data from publicly available microarray databases. In fact, the absence of statistically significant heterogeneity and bias in the meta-analysis are indicators of the sufficient quality of the meta-analyses and the validity of the deriving conclusions.

Description of FcRn expression in a small set of patients, by IHC, revealed a marked expression by alveolar macrophages and a faint signal from epithelial bronchial cells in the normal lungs, as previously described [20]. In NSCLC, FcRn expression was mainly found in infiltrating immune cells, in particular macrophages and DCs. Further studies will be required to assess if *FCGRT* mRNA reduction is attributed to a lower density of FcRn^+^-cells or/and to molecular mechanisms underlying mRNA down-regulation in these cells. In colorectal carcinoma, Baker et *al*. [18] found that FcRn-positive dendritic cells (DCs) were strongly correlated with the presence of CD8^+^ T cells in the non-cancerous tissue and promote the anti-tumor role of FcRn. In NSCLC, the global expression of *FCGRT* mRNA might be a reflection of the density of antigen presenting cells and antitumor immune response in NSCLC. In that context, it will be interesting to evaluate the predictive value of FcRn in response to immunomodulatory monoclonal antibodies, targeting immune checkpoints (anti-PD-1 or anti-CTLA-4) that have raised great promises in the treatment of NSCLC [30, 31].

Although the recommended tumor, node and metastasis (TNM) classification and stage determination are important to select therapeutic options for patients with non-small cell lung carcinoma (NSCLC), additional molecular markers are required to indicate better the prognosis, in particular within a specific stage, and determine the potential benefits of adapted therapy in NSCLC [32]. We report the first demonstration of the prognostic and predictive values of testing for *FCGRT* mRNA in NSCLC patients. The prognostic value of testing for FcRn in specific cell populations by double IHC staining has already been described in colorectal carcinoma [18]. Although our study suffers from the common limitations of investigations with surgical tumor samples, being mainly descriptive, as well as a retrospective nature and a certain degree of heterogeneity of the samples, our results identified *FCGRT* mRNA level as a robust and independent marker of NSCLC patient outcome and add to the existing evidences on the central role of the tumor's niche on carcinogenesis and disease progression [33]. Future external validations, in one or more independent, ideally multicentric, prospective studies would be required to support our findings and help transfer our results to a clinical application for NSCLC.

Testing for *FCGRT* mRNA is straightforward, and can readily be applied in the clinic. This novel marker could be combined with the recently described Immunoscore® [34], a method measuring the beneficial impact of the immune infiltrate on tumor outcome, which has emerged in colorectal cancer and may be relevant in other malignancies, such as lung cancer [35]. Taking together, they may help with decision-making for NSCLC management, contributing to the timely and appropriate administration of adjuvant treatment.

## MATERIALS AND METHODS

### Lung tissue specimens

Specimens (2-3 cm^2^) of cancerous and non-cancerous tissues (80 patients) were collected from patients prior to any therapy, as described by Gueugnon et *al.* [36] at the Trousseau Hospital, Tours, France, between 2006 and 2011 (n° DC-2008-308 – also named in the manuscript Tours' hospital cohort). The nonmalignant tissue samples were taken from sites at least 3 cm away from the edge of the tumor. Patient data were recorded in a database for statistical analysis (See Tables 4 and 5). This study was conducted in accordance with the ethical standards of the Helsinki Declaration and French bioethical authorities. Frozen lung tissues were embedded in Tissue-Tek OCT (Sakura Finetek Europe) and 8μm sections were cut on a cryotome (Thermo SCIENTIFIC). Sections (up to 50 mg) were immediately placed in lysis buffer (RLT Plus, Qiagen) containing 143 μM β-mercaptoethanol and vortexed thoroughly for 2 minutes. The lysates were then centrifuged in a microfuge for 1 min at full speed. Aliquots of each supernatant were collected and stored at −80°C. RNA was purified on a QIAsymphony SP workstation using the RNA CT 400 protocol (QIAsymphony RNA kit, Qiagen), including digestion of genomic DNA with DNAse I. Total RNA was eluted RNAse-free water and quantified using a NanoDrop 2000c spectrophotometer (Thermo SCIENTIFIC). RNA integrity was assessed using an Agilent 2100 bioanalyzer. Only samples with an RNA integrity number (RIN) > 6 were considered for RT-qPCR analysis (80 patients matched for tumoral and non tumoral adjacent tissue).

**Table 4:** Patient characteristics (n=80)

Characteristics	N (%)
**Gender**	
Male	64 (80)
Female	16 (20)
**Smoking history**	
Never	9 (11.25)
Former/Current	68 (85)
Unknown	3 (3.75)
**Histologic type**	
Squamous cell carcinoma (SCC)	35 (43.75)
Adenocarcinoma (ADC)	45 (56.25)
**T- primary tumor size**	
≤ 3cm	27 (33.75)
> 3cm	48 (60)
Unknown	5 (6.25)
**N- lymph node status**	
Yes	47 (58.75)
No	31 (38.75)
Unknown	2 (2.5)
**M- distant metastasis**	
Yes	13 (16.25)
No	65 (81.25)
Unknown	2 (2.5)
**pTNM Stage**	
IA	11 (13.75)
IB	26 (32.5)
IIA	5 (6.25)
IIB	8 (10)
IIIA	13 (16.25)
IIIB	3 (3.75)
IV	13 (16.25)
Unknown	1 (1.25)

**Table 5:** Descriptive statistics of the continuous variables (n=80)

	Percentiles									
	N	Mean	S.E.	Min	Max	10	25	50 (median)	75	90
**Age**	80	65.2	1.2	36.0	83.0	50.2	59.0	66.5	73.0	78.0
**Tumor size (cm)**	75	3.75	0.18	1.20	10.0	2.00	2.50	3.50	4.50	6.00
**Packs per year**	66	42.8	2.4	5.0	100	20.0	30.0	40.0	50.0	75.0
**Smoking cessation (years)**	57	7.32	1.50	0.0	60.0	0.0	0.0	0.0	11.5	22.4
**OS (months)**	71	30.0	2.14	3.0	78.0	7.0	17.0	27.0	42.0	58.6

Single-strand cDNA was synthesized from 2μg total RNA from each sample with the High Capacity cDNA Reverse Transcription kit (Applied Biosystems), according to the manufacturer's instructions.

### Quantitative real-time PCR

Following RNA extraction from NSCL and non-cancerous adjacent tissues, and cDNA synthesis, quantitative-PCR was carried out on the LightCycler 480 (Roche Diagnostics GmbH) as described by Gueugnon et *al.* [36]. The concentration of *FCGRT* mRNA was normalized to the geometric mean of mRNAs of two reference genes: TATA-binding protein (*TBP*) and hypoxanthine phosphoribosyltransferase 1 (*HPRT1*). This gives more reliable results than using a single reference gene [37]. Gene-specific primer pairs were designed according published mRNA sequences so that the amplicons generated spanned two exons. The sequences of the primers used in this study are: *FCGRT* F 5′-CCCTGGCTTTTCCGTGCTT-3′; R 5′-TGACGATTCCCACCACGAG-3′; *HPRT1* F 5′-CAT TATGCTGAGGATTTGGAAGG-3′; R 5′-CTTGAGCAC ACAGAGGGCTACA-3′; TBP F 5′-TGTATCCACAG TGAATCTTGGT TG-3′; R 5′-GGTTCGTGGCTCTCT TATCCTC-3′. PCR reactions were carried out using 20ng cDNA as template, 0.2μM each of forward and reverse primer and 1x SYBR Premix Ex Taq (Takara Bio Inc). Each reaction was performed in triplicate. The thermal protocol consisted of an initial denaturation step at 95°C for 30 sec followed by 40 cycles of denaturation at 95°C for 5 sec and primer annealing and extension at 60°C for 20 sec. Melting curves were generated for each amplified cDNA to check the specificity of the reactions. In the standard protocol, fluorescence was read at 60°C during the annealing and extension step. However, we included an additional step (heating at 82°C for 15sec) to record the fluorescence. This increased the specificity of *FCGRT* measurement by eliminating all non-specific signals. Each PCR run included a no-template control and a calibrator AV090211 (a pool of cDNA from many lung tumors) to evaluate inter-assay variability.

Serial dilutions of the AV090211 calibrator (a pool of 10 cDNA from tumoral and non tumoral samples) (80ng cDNA to 0.625ng cDNA) were used to create a standard curve for each gene. These curves were constructed by plotting the crossing point (Cp) values against the initial quantity of the AV090211 calibrator. The Cp of a sample, defined as the point where the fluorescence curve of the sample was above the background fluorescence, was calculated according to the second derivative maximum method by the LightCycler 480 software. The efficiencies of PCR for t *FCGRT*and reference genes were calculated from the formula E = 10^−1/slope^; they were close to 100% (i.e. efficiency E = 2): *FCGRT*: E = 1.986; *TBP*: E = 2.041; *HPRT1*: E = 1.979. Thus, the software converted Cp data from the samples into a concentration for each gene using the PCR efficiency and the initial amount of AV090211 calibrator. Finally, the results for each sample were normalized by dividing the *FCGRT* value by the geometric mean of *HPRT1* and *TBP* reference genes.

### Western blot

Protein extracts (40μg) of a pool of 10 cancerous tissues and 10 matched non-cancerous tissues from NSCLC patients, chosen blindly and human recombinant FcRn protein were separated on a NUPAGE 4-12% Bis-Tris gel (Life Technologies) and then transferred onto PVDF (polyvinyldenedifluoride) membranes by electroblotting. Membranes were blocked and then incubated with anti-FcRn (1/500) (Novus Biologicals cat. number NBP1-89128) or anti-alpha tubulin (1/20000) as a loading control antibody (Abcam cat. number ab7291) under the conditions recommended by the manufacturers. After incubation with the appropriate conjugated-HRP secondary antibody, membranes were developed using an enhanced chemiluminescence western blotting detection reagent (Amersham Biosciences). Densitometry of the FcRn spots was quantified using ImageJ and normalized with α-tubulin. The experiments were done three times with two different pools of proteins from 10 patients. The FcRn human recombinant protein used as a positive control is homologous over its entire region to the amino acid sequence PAKS, from there it is deleted on the entire Ct final region and the original sequence was replaced by a synthetic sequence which corresponds to the sequence factor X, followed by the V5 epitope and a polyhistidine. The construction has been obtained by a 2-step cloning 1) a deletion from PAKS (since the beginning of exon 6) then 2) an insertion of the synthetic sequence at BamHI-NotI site.

### Immunohistochemical analysis

Staining of FcRn^+^- cells, dendritic cells, macrophages and CD8^+^-T cells was assessed on both cancerous (C) and non-cancerous (NC) 3μm-thick serial lung tissue sections from a small set of patients (n=8), using anti-FcRn (Abcam cat. number ab4360), anti-PS100 (Dako, cat. number Z0311), anti-CD163 (LEICA, cat. number NCL-CD163) and anti-CD8 (Dako, cat. number M7103) antibodies, respectively. Briefly, tissues were deparaffinized, rehydrated and subjected to heat antigen retrieval in a citrate buffer (pH 6.0). FcRn staining was done as followed: samples were washed twice in TBS-Tween 0.1% before being blocked for endogenous peroxidase activity in 3% hydrogen peroxide-methanol. After 2 washes, the VECTASTAIN Elite ABC Kit (Goat IgG, VECTOR Laboratories) was used for immunostaining following the manufacturer's instructions. Tissues sections were blocked 1h in blocking buffer (*PBS-triton 0.4% + 3 drops of goat sera (from the VECTASTAIN Elite ABC kit)* + 5% BSA + 3% normal human sera buffer). Tissue sections were incubated with anti-FcRn (diluted 1/500) polyclonal antibody (Novus Biologicals cat. number NBP1-89128) overnight at 4°C in blocking buffer. A standard avidin–biotin immunoperoxidase method and diaminobenzidine as chromogen (DakoCytomation) were used for visualization. Tissues slides were colored using Harris' hematoxylin. Rabbit IgG (1/28,000), whole molecule (Jackson ImmunoResearch), was used as both isotype and negative control. Positive and negative controls were realized on human placenta section with FcRn antibody. PS100, CD163 and CD8 staining were done using a benchmark XT automated stainer (Roche) following the manufacturer's instructions; tissues sections were colored with Gill's hematoxylin.

### Statistical analyses

Differences between *FCGRT* expression values between groups of cancerous samples were assessed using the non-parametric Mann-Whitney U and Jonckheere-Terpstra tests. *FCGRT* mRNA levels in paired NSCLC samples were compared with the non-parametric Wilcoxon signed-rank test. The DeLong *et al*. [38] method was used for ROC curve analyses. The optimal cut-off that derived from the ROC curve analysis corresponded to the highest accuracy as calculated by the Youden index J.

Kaplan-Meier survival curves were analyzed for the overall survival of the 71 NSCLC patients with available follow-up data; the log-rank test was used for evaluation of statistical significance. Cox regression models were used to evaluate the prognostic potential of *FCGRT* mRNA levels for overall survival (OS) of NSCLC patients through the calculation of hazard ratios (HR) at the univariate and multivariate levels. Multivariate models were adjusted for tumor stage, tumor histotype and patient age (model a) or metastasis status, tumor histotype and patient age (model b). For survival analyses, optimal cut-off points were established using the X-tile algorithm and the minimal p-value approach for cutoff optimization. All regression models were validated by the bootstrap resampling (n=1,000 bootstrap samples) using the bias corrected and accelerated approach. The selected cut-off for FcRn expression in the cancerous tissue parts was additionally validated (P=0.0197) in the Kaplan-Meier Plotter (KM Plotter) database, indicating that it can be applied in an independent dataset. SPSS Statistics (version 17.0), MedCalc software (version 12.5) and GraphPad Prism (version 5.00) were used.

### Publicly available databases, online survival analysis tools and meta-analysis

The Oncomine database and gene microarray analysis tool, a repository for published cDNA microarray data (www.oncomine.org) [23] was used (January 2015) to analyze the *FCGRT* mRNA expression in public Lung cancer datasets [39–42]. Oncomine algorithms were used for the statistical analysis of the differences in FCGRT mRNA expression and association with patient survival status (Student's t-test).

The Kaplan Meier-Plotter (KM Plotter) online survival analysis tool was used in order to confirm the prognostic potential of FcRn in a large cohort of lung cancer patients. Briefly, gene expression data and overall survival information are downloaded from GEO, EGA and TCGA and a database is created. To analyze the prognostic value of a particular gene, in our case *FCGRT*, the patient samples are split into two groups according to various quantile expressions and are compared by a Kaplan-Meier survival plot. KM plotter was used both at the univariate and multivariate level [24].

Meta-analysis on FcRn prognostic significance was performed by gathering all available Hazard Ratios and 95% Confidence Intervals for FcRn in lung cancer from different individual microarray-based studies using the publicly available biomarker analysis and validation tools: Prognoscan [25], ProgGene V2 [26], SurvExpress [27] and KM Plotter [24]. Subsequently we analyzed this information with the StatsDirect v3.0 [StatsDirect Ltd. StatsDirect statistical software. http://www.statsdirect.com. England: StatsDirect Ltd. 2013]. Statistical software in order to calculate the pooled Hazard Ratio, Cochran Q and I^2^ values, as well as and to produce Forest plots and Bias-assessment plots.

## SUPPLEMENTARY MATERIALS AND METHODS


