# Supplementary material for: Downregulation of the neonatal Fc receptor expression in non-small cell lung cancer tissue is associated with a poor prognosis
Source: Oncotarget. 2016 Jun 15;7(34):54415–29. doi: 10.18632/oncotarget.10074 (PMC5342352; doi:10.18632/oncotarget.10074)
Supplement: Supplementary file 1 [file oncotarget-07-54415-s001.pdf]

Downregulation of the neonatal Fc receptor expression in non-small cell lung cancer tissue is associated with a poor prognosis

SUPPLEMENTARY MATERIALS AND METHODS

Total RNA isolation and cDNA synthesis

NSCLC and non tumoral adjacent tissues

Frozen lung tissues were embedded in Tissue-Tek OCT (Sakura Finetek Europe) and 8µm sections were cut on a cryotome (Thermo SCIENTIFIC). Sections (up to 50 mg) were immediately placed in lysis buffer (RLT Plus, Qiagen) containing 143 µM β-mercaptoethanol and vortexed thoroughly for 2 minutes. The lysates were then centrifuged in a microfuge for 1 min at full speed. Aliquots of each supernatant were collected and stored at -80°C. RNA was purified on a QIAsymphony SP workstation using the RNA CT 400 protocol (QIAsymphony RNA kit, Qiagen), including digestion of genomic DNA with DNAse I. Total RNA was eluted RNAse-free water and quantified using a NanoDrop 2000c spectrophotometer (Thermo SCIENTIFIC). RNA integrity was assessed using an Agilent 2100 bioanalyzer. Only samples with an RNA integrity number (RIN) > 6 were considered for RT-qPCR analysis (80 patients matched for tumoral and non tumoral adjacent tissue).

Single-strand cDNA was synthesized from 2µg total RNA from each sample with the High Capacity cDNA Reverse Transcription kit (Applied Biosystems), according to the manufacturer's instructions.

CRC and non tumoral adjacent tissues

All tissue samples were maintained at 180°C (liquid nitrogen) until RNA extraction and were weighed before homogenization. Tissue samples were then disrupted directly into a lysis buffer using Mixer Mill MM 300 (Qiagen). Total RNA was isolated from tissue lysates using the RNeasy Mini Kit (Qiagen), and additionalDNAsedigestion was performed on all samples during the extraction process (RNase-Free DNase Set Protocol for DNase treatment on RNeasy Mini Spin Columns; Qiagen). After each extraction, a small fraction of the total RNA preparation was taken to determine the quality of the sample and the yield of total RNA. Controls analyses were performed by UV spectroscopy and analysis of total RNA profile using the Agilent RNA 6000 Nano LabChip Kit with the Agilent 2100 Bioanalyser (Agilent Technologies) to determine RNA purity, quantity, and integrity.

Quantitative real-time PCR

The *FCGRT* mRNA expression in lung samples was determined by quantitative real-time PCR in a LightCycler 480 (Roche Diagnostics GmbH). The concentration of *FCGRT* mRNA was normalized to the geometric mean of mRNAs of two reference genes. TATA-binding protein (*TBP*) and hypoxanthine phosphoribosyltransferase 1 (*HPRT1*). This gives more reliable results than using a single reference gene (Vandesompele et al. 2002). Gene-specific primer pairs were designed according published mRNA sequences so that the amplicons generated spanned two exons. The sequences of the primers used in this study are:

Gene	Forward	Reverse
FCGRT	5'-CCCTGGCTTTTCCGTGCTT-3'	5'-TGACGATTCCCACCACGAG-3'
HPRT1	5'-CATTATGCTGAGGATTTGGA AGG-3'	5'-CTTGAGCACACAGAGGGCT ACA-3'
TBP	5'-TGTATCCACAGTGAATCTTGG TTG-3'	5'-GGTTCGTGGCTCTCTTATC CTC-3'

PCR reactions were carried out using 20ng cDNA as template, 0.2µM each of forward and reverse primer and 1x SYBR Premix Ex Taq (Takara Bio Inc). Each reaction was performed in triplicate. The thermal protocol consisted of an initial denaturation step at 95°C for 30 sec followed by 40 cycles of denaturation at 95°C for 5 sec and primer annealing and extension at 60°C for 20 sec. Melting curves were generated for each amplified cDNA to check the specificity of the reactions. In the standard protocol, fluorescence was read at 60°C during the annealing and extension step. However, we included an additional step (heating at 82°C for 15sec) to record the fluorescence. This increased the specificity of *FCGRT* measurement by eliminating all non-specific signals. Each PCR run included a no-template control and a calibrator AV090211 (a pool of cDNA from many lung tumors) to evaluate inter-assay variability.

Serial dilutions of the AV090211 calibrator (a pool of 10 cDNA from tumoral and non tumoral samples) (80ng cDNA to 0.625ng cDNA) were used to create a standard curve for each gene. These curves were constructed by plotting the crossing point (Cp) values against the initial quantity of the AV090211 calibrator. The Cp of a sample, defined as the point where the fluorescence curve of the

sample was above the background fluorescence, was calculated according to the second derivative maximum method by the LightCycler 480 software. The efficiencies of PCR for the target and reference genes were calculated from the formula $E = 10^{-1/slope}$; they were close to 100% (i.e. efficiency E = 2): *FCGRT*: E = 1.986; *TBP*: E = 2.041; *HPRT1*: E = 1.979. Thus, the software converted Cp data from the samples into a concentration for each gene using the PCR efficiency and the initial amount of AV090211 calibrator. Finally, the results for each sample were normalized by dividing the *FCGRT* value by the geometric mean of *HPRT1* and *TBP* reference genes.

Western blot

Protein extracts (40µg) of a pool of 10 cancerous tissues and 10 matched non-cancerous tissues from NSCLC patients, chosen blindly and human recombinant FcRn protein were separated on a NUPAGE 4-12% Bis-Tris gel (Life Technologies) and then transferred onto PVDF (polyvinyldenedifluoride) membranes by electroblotting. Membranes were blocked and then incubated with anti-FcRn (Novus Biologicals) or anti-alpha tubulin as a loading control antibody (Abcam) under the conditions recommended by the manufacturers. After incubation with the appropriate conjugated-HRP secondary antibody, membranes were developed using an enhanced chemiluminescence western blotting detection reagent (Amersham Biosciences). The FcRn human recombinant protein used as a positive control is homologous over its entire region to the amino acid sequence PAKS, from there it is deleted on the entire Ct final region and the original sequence was replaced by a synthetic sequence which corresponds to the sequence factor X, followed by the V5 epitope and a polyhistidine (10). The construction has been obtained by a 2-step cloning 1) a deletion from PAKS (since the beginning of exon 6) then 2) an insertion of the synthetic sequence at BamHI-NotI site.

Immunohistochemical analysis

10µm-thick tissue sections from both NSCLC and non-cancerous tissues were deparaffinized, rehydrated and subjected to heat antigen retrieval in a citrate buffer (pH 6.0). Samples were blocked for endogenous peroxidase activity in 3% hydrogen peroxide-methanol. The VECTASTAIN Elite ABC Kit (Goat IgG,VECTOR Laboratories) was used for immunostaining following the manufacturer's instructions. Tissue sections were incubated with anti-FcRn polyclonal antibody (Novus Biologicals) overnight at 4°C. A standard avidin–biotinimmunoperoxidase method and diaminobenzine as chromogen (DakoCytomation) were used for visualization. Rabbit IgG, whole molecule (Jackson ImmunoResearch), was used as a negative control.

SUPPLEMENTARY FIGURE AND TABLES

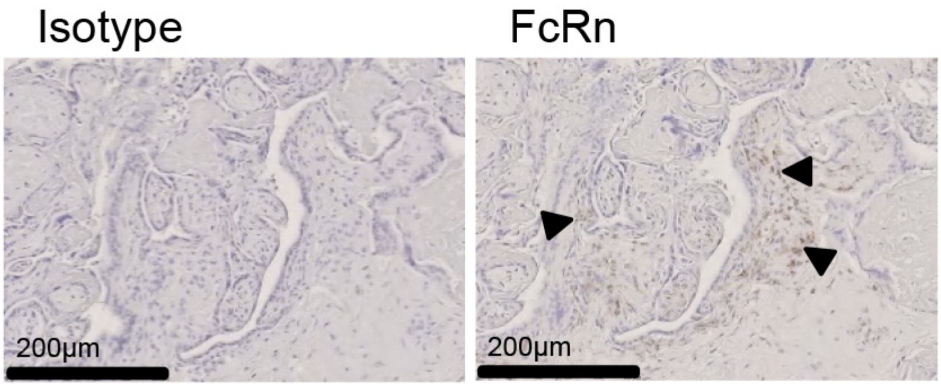

Supplementary Figure S1: Negative (Isotype: Rabbit IgG whole molecule) and positive controls of FcRn immunostaining were realized on commercial human placenta slides. No staining was observed with isotype whereas arrowheads indicate FcRn staining (in brown).

Supplementary Table S1: Associations between *FGRT* mRNA levels and clinicopathological characteristics of NSCLC patients

	N	Median FCRN expression in NSCLC	P value
**Gender**			
Male	64	0.449	0.085[a]
Female	16	0.698	
**Histotype**			
Squamous cell carcinoma	35	0.380	0.091[a]
Adenocarcinoma	45	0.556	
**Differentiation status**			
Moderately or well	34	0.387	0.501[a]
Poorly	15	0.470	
Unknown	31		
**Tumor size**			
≤3 cm	27	0.487	0.843[a]
>3 cm	48	0.496	
Unknown	5		
**Lymph node status**			
Negative	47	0.532	0.755[a]
Positive	31	0.451	
Unknown	2		

(*Continued*)

	N	Median FCRN expression in NSCLC	P value
**Metastasis**			
No	65	0.522	0.507[a]
Yes	13	0.382	
Unknown	2		
**Stage**			
I, II	50	0.496	0.692[a]
III, IV	29	0.477	
Unknown	1		
**Chronic Obstructive Pulmonary Disease**			
No	44	0.558	0.246[a]
Yes	36	0.409	
**Smoking status**			
Never	9	0.810	0.234[a]
Currently or former	68	0.455	
Unknown	3		
**Pack-years**			
<21	9	0.579	0.179[b]
21-49	34	0.464	
>49	23	0.407	
Unknown	14		
**Stage (ordinal)**			
Ia	11	0.823[c]	0.144[b]
Ib	26	0.432[c]	
IIa	5	0.314[d]	
IIb	8	0.382[d]	
IIIa	13	0.487[e]	
IIIb	3	0.810[e]	
IV	13	0.382[e]	
Unknown	1		

[a] P value calculated by Mann-Whitney test
[b] P value calculated by Jonckheere-Terpstra test
[c]P = 0.043 (for Stage Ia *vs* Stage Ib, Mann-Whitney test)
[d]P = 0.380 for (for Stage IIa *vs* Stage IIb, Mann-Whitney test)
[e]P = 0.726 for (for Stage III *vs* Stage IV, Mann-Whitney test)

Supplementary Table S2: The prognostic value of *FCGRT* mRNA levels for early stage and metastasis-free NSCLC patients (Kaplan-Meier analysis)

Variable	Mean overall survival time in months (95% CI)	P*
**Early stage (I/II) patients (n=45)**		
***FCGRT* mRNA level in cancerous tissue parts**		
Low (l) (n=34)	41.3 (32.7 – 49.8)	0.339
High (h) (n=11)	61.1 (44.8 – 77.4)	
***FCGRT* mRNA level in non-cancerous tissue parts**		
Low (n=20)	41.1 (26.4 – 55.8)	**0.035**
High (n=25)	50.5 (42.3 – 58.8)	
**Combined *FCGRT* mRNA levels**		
C^l / NC^l (n=15)	31.1 (18.8 – 43.5)	**0.055**
C^h and/or NC^h (n=30)	55.8 (44.8 – 66.7)	
**Non-metastatic patients (n=57)**		
***FCGRT* mRNA level in cancerous tissue parts**		
Low (n=44)	40.0 (32.7 – 47.3)	0.108
High (n=13)	63.8 (49.7 – 77.9)	
***FCGRT* mRNA level in non-cancerous tissue parts**		
Low (n=23)	36.8 (23.6 -50.0)	**0.007**
High (n=34)	54.1(45.6 – 62.6)	
**Combined *FCGRT* mRNA levels**		
C^l / NC^l (n=18)	29.9 (18.9 – 41.0)	**0.008**
C^h and/or NC^h (n=39)	56.1 (46.6 – 65.6)	
**Late stage (III/IV) patients (n=26)**		
***FCGRT* mRNA level in cancerous tissue parts**		
Low (l) (n=21)	31.0 (22.0 – 40.0)	0.080
High (h) (n=5)	58.3 (38.1 – 78.6)	
***FCGRT* mRNA level in non-cancerous tissue parts**		
Low (n=10)	25.8 (11.7 – 39.9)	0.076
High (n=16)	45.1 (31.8–58.4)	
**Combined *FCGRT* mRNA levels**		
C^l / NC^l (n=8)	21.1 (5.8–36.5)	**0.024**
C^h and/or NC^h (n=18)	44.7 (32.3 – 57.1)	
**Metastatic patients (n=12)**		
***FCGRT* mRNA level in cancerous tissue parts**		
Low (l) (n=9)	20.4 (10.5 – 30.4)	0.110
High (h) (n=3)	34.5 (32.4 – 36.6)	

(*Continued*)

Variable	Mean overall survival time in months (95% CI)	P*
***FCGRT* mRNA level in non-cancerous tissue parts**		
Low (n=6)	18.2 (8.2 – 28.1)	0.272
High (n=6)	31.5 (18.8 – 44.2)	
**Combined *FCGRT* mRNA levels**		
C^l / NC^l (n=4)	10.0 (3.7– 16.4)	**0.003**
C^h and/or NC^h (n=8)	32.4 (22.5 – 42.3)	

*P value calculated by the log-rank test (Kaplan-Meier survival analysis).

Supplementary Table S3: Multivariate analysis for the association of *FCGRT* mRNA expression and overall survival (adjusted for Stage and Histology), based on the KM-plotter platform (n=890 available patients).

	P value	Hazard Ratio
Histology	0.0002	1.38 (1.17 - 1.64)
Stage	0	1.49 (1.3 - 1.7)
FCGRT	**0.0009**	**0.69 (0.55 - 0.86)**

Supplementary Table S4: Data used for the meta-analysis and construction of Forest Plot for NSCLC (Figure 4 B-C)

Study	HR	Low	High	P value	SE	Weights (fixed)	Weights (random)
jacob-00182-CANDF	0.56	0.35	0.88	0.0129397	0.235	2.083	2.499
HARVARD-LC	0.46	0.29	0.74	0.00120089	0.239	2.018	2.425
jacob-00182-HLM	0.56	0.36	0.87	0.0105452	0.225	2.274	2.711
MICHIGAN-LC	0.25	0.09	0.7	0.00826201	0.523	0.421	0.533
jacob-00182-MSK	0.77	0.45	1.35	0.364188	0.280	1.467	1.795
GSE13213	0.63	0.41	0.98	0.0394138	0.222	2.332	2.775
GSE31210	1.22	0.45	3.31	0.702288	0.509	0.445	0.563
jacob-00182-UM	0.79	0.5	1.26	0.325736	0.236	2.073	2.487
GSE11117	0.6	0.37	0.98	0.0405953	0.248	1.866	2.254
GSE3141	1.07	0.74	1.55	0.71182	0.189	3.239	3.747
GSE14814	0.95	0.54	1.67	0.868149	0.288	1.389	1.704
GSE4716-GPL3696	0.29	0.09	0.95	0.0408259	0.601	0.319	0.405
GSE4573	0.83	0.57	1.22	0.353167	0.194	3.058	3.557
GSE17710	0.74	0.44	1.24	0.252865	0.264	1.650	2.006

(*Continued*)

Study	HR	Low	High	P value	SE	Weights (fixed)	Weights (random)
GSE5843	0.89	0.4	1.96	0.7728	0.405	0.701	0.880
GSE11969	0.15	0.02	1.91	0.1456	1.163	0.085	0.109
GSE26939	0.73	0.49	1.09	0.1285	0.204	2.770	3.251
GSE17710	0.65	0.39	1.07	0.0919	0.257	1.738	2.108
GSE19188	0.78	0.49	1.23	0.2813	0.235	2.091	2.507
GSE41271	0.69	0.56	0.84	0.0003	0.103	10.771	10.114
KM Plotter database	0.69	0.6	0.79	0.00000008	0.070	23.399	16.700
CAARAY	0.61	0.48	0.79	0.0001	0.127	7.133	7.368
GSE29013	1.48	0.57	3.81	0.419	0.485	0.491	0.620
GSE30219	0.75	0.54	1.04	0.084	0.167	4.122	4.642
GSE31908	2.06	0.41	10.28	0.3672	0.822	0.171	0.218
GSE37745	0.5	0.35	0.7	0.00004	0.177	3.686	4.206
GSE50081	0.71	0.43	1.17	0.17	0.255	1.767	2.141
TCGA	0.54	0.24	1.21	0.13	0.413	0.677	0.850
SurvExpress database	0.74	0.61	0.88	0.001096	0.093	13.186	11.677
GSE5123	0.74	0.34	1.59	0.4374	0.394	0.744	0.932
GSE42127	0.85	0.52	1.39	0.5014	0.251	1.832	2.215
